# Melioidosis Caused by *Burkholderia pseudomallei* in Drinking Water, Thailand, 2012

**DOI:** 10.3201/eid2002.121891

**Published:** 2014-02

**Authors:** Direk Limmathurotsakul, Gumphol Wongsuvan, David Aanensen, Sujittra Ngamwilai, Natnaree Saiprom, Patpong Rongkard, Janjira Thaipadungpanit, Manas Kanoksil, Narisara Chantratita, Nicholas P.J. Day, Sharon J. Peacock

**Affiliations:** Mahidol University, Bangkok, Thailand (D. Limmathurotsakul, G. Wongsuvan, S. Ngamwilai, N. Saiprom, P. Rongkard, J. Thaipadungpanit, N. Chantratits, N.P.J. Day, S.J. Peacock);; Imperial College London, London, UK (D. Aanensen);; Sappasithiprasong Hospital, Ubon Ratchathani, Thailand (M. Kanoksil);; Churchill Hospital, Oxford, UK (N.P.J. Day);; Addenbrooke’s Hospital, Cambridge, UK (S.J. Peacock)

**Keywords:** melioidosis, Burkholderia pseudomallei, bacteria, drinking water, genotyping, Thailand

## Abstract

We identified 10 patients in Thailand with culture-confirmed melioidosis who had *Burkholderia pseudomallei* isolated from their drinking water. The multilocus sequence type of *B. pseudomallei* from clinical specimens and water samples were identical for 2 patients. This finding suggests that drinking water is a preventable source of *B. pseudomallei* infection.

*Burkholderia pseudomallei* is a Tier 1 select agent and the cause of naturally acquired melioidosis in Southeast Asia, northern Australia, the Indian subcontinent, and areas of South America (*1*). The organism is present in soil and surface water, and most melioidosis cases are believed to result from bacterial inoculation or inhalation (*1*). Ingestion has been increasingly suspected to be an alternative route of infection. *B. pseudomallei* isolated from a community water supply in Australia was genetically identical to that causing disease in clusters (*2*,*3*), although no direct evidence was available to show that affected cases had consumed contaminated water. The pattern of infection after ingestion in an experimental model includes multiple organ dissemination and hepatosplenic abscesses, which are common features of human melioidosis and supportive evidence for ingestion as a route of human infection (*4*).

Our previous study in Ubon Ratchathani Province in northeastern Thailand investigated the activities of daily living associated with acquisition of melioidosis (*5*). Households of participants who resided ≤100 km of Sappasithiprasong Hospital in Ubon Ratchathani were visited, and water samples were collected from all sources of drinking water and from tap water (*5*). Culture of these samples for *B. pseudomallei* provided borderline statistical evidence to suggest that consuming water containing *B. pseudomallei* was associated with melioidosis (conditional odds ratio 2.2, 95% CI 0.8–5.8, p = 0.08) (*5*). We performed a study to further evaluate the role of ingestion as a route of infection, and we identified the genotypes of *B. pseudomallei* isolated from patients and the water supplies consumed by them.

## The Study

During July 2010–December 2011, we collected and cultured 576 water samples from the households of 142 case-patients and 288 controls (*5*). In brief, 5 L of water was collected from each source of drinking water and tap water, regardless of consumption. If the water was filtered or boiled by the householder before consumption, samples were collected for culture. Locations from which water samples were collected were recorded by using the Epicollect Program (*6*). For each sample, 1 L was passed through two 0.45-μm filters (500 mL through each filter), and the remaining 4 L was passed through 2.5 g of sterile diatomaceous earth (Celite; World Minerals Corporation, San Jose, CA, USA).

Filters were cultured on Ashdown agar to obtain a quantitative bacterial count, and diatomaceous earth was cultured in selective broth containing (15 mL of threonine–basal salt plus colistin broth) to obtain a sensitive, qualitative method. Broth was incubated at 40°C in air for 48 h, after which 10 μL of the upper layer was streaked onto an Ashdown agar plate to achieve single colonies, incubated at 40°C in air, and examined every 24 h for 7 days. If enrichment broth cultures showed positive results but filters on Ashdown agar showed negative results, the quantitative count was defined as <1 CFU/L. Ten colonies of *B. pseudomallei* were randomly picked from the primary plate and saved for genotyping. If there were <10 colonies, all primary plate colonies were used, and the number was adjusted to 10 after subculture of the enrichment broth.

Genotyping was performed by using pulsed-field gel electrophoresis and multilocus sequence typing (MLST) as described (*7*,*8*). In brief, isolates from clinical and water samples from the same patient were subjected to electrophoresis on the same gel. Colonies with an identical banding pattern were classified as the same genotype, and colonies with ≥1 different patterns were further genotyped by using MLST (*8*). A map was drawn by using the R program (www.r-project.org/) and OpenStreetMap (www.openstreetmap.org) data.

**Figure 1:**
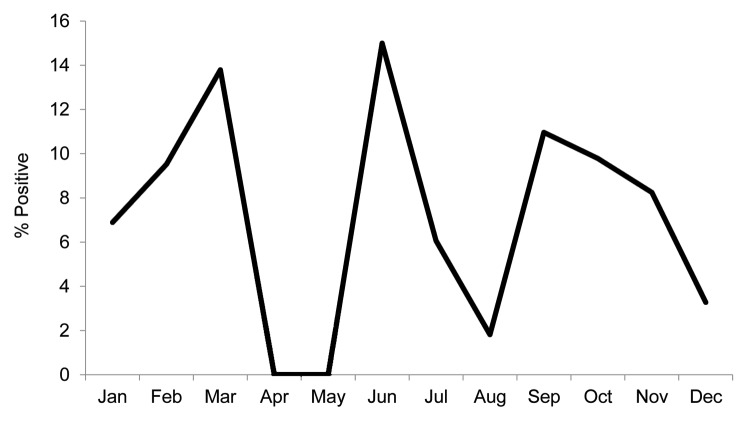
Percentage of water samples positive for *Burkholderia pseudomallei*, Thailand, 2012.

**Figure 2:**
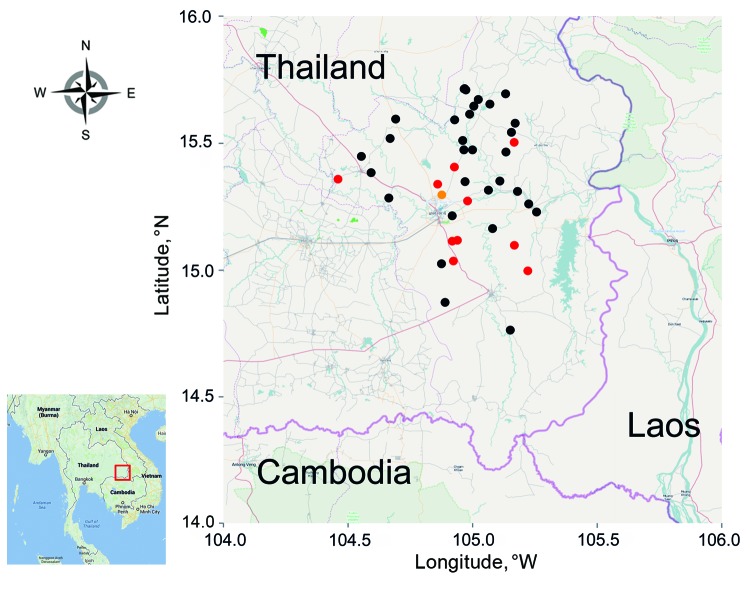
Ubon Ratchathani Province in northeastern Thailand and locations where water samples were tested for *Burkholderia pseudomallei*, 2012. Location of wells, bore holes, and tap water samples that were positive are indicated by orange, red, and black circles, respectively. The red square in the inset indicates the study area in Thailand.

A total of 43 (7%) of 576 water samples were culture positive for *B. pseudomallei* (Table 1). The rate of positivity did not differ between the rainy season (June–November) and dry season (December–May) (8% vs. 6%; p = 0.32, by Fisher exact test) (Figure 1). Positive water samples were geographically distributed across the sampling area (Figure 2). The median quantitative count of *B. pseudomallei* in water was 1 CFU/L (interquartile range <1–13 CFU/L, range <1–65 CFU/L) (Table 1). Of the 43 culture-positive water samples, 21 (7%) of 288 were from control households and 22 (15%) of 142 were from case-patient households. Ten of these case-patients with melioidosis reported drinking from contaminated water sources in the 30 days before the onset of illness.

**Table 1:** Culture of *Burkholderia pseudomallei* from household drinking water in Ulbon Ratchathani, Thailand, 2012*

Source of drinking water†	No. positive samples/no. tested (%)	Median quantitative count of *B. pseudomallei*, CFU/L (range)
Well	1/27 (4)	31
Bore hole	10/84 (12)	18 (<1–65)
Collected rain	0/160 (0)	NA
Tap		
Available but not consumed	26/178 (15)	<1 (<1–63)
Consumed	6/95 (6)	1 (<1–13)
Bottled	0/32 (0)	NA
Total	43/576 (7)	1 (<1–65)

*Households of participants who resided ≤100 km of Sappasithiprasong Hospital in Ubon Ratchathani were visited. NA, not applicable. †Samples from water that was consumed were collected, together with tap water samples, which were collected regardless of ingestion history.

We compared genotypes of *B. pseudomallei* in clinical specimens and water samples from the 10 case-patients who consumed water that was subsequently shown to contain *B. pseudomallei*. A total of 91 colonies from 10 water samples and 1 colony isolated from blood culture (*7*) or sputum (*3*) from each case-patient was examined by using pulsed-field gel electrophoresis and MLST. The median number of different genotypes observed per water sample was 3 (range 1–6). Two case-patients were infected with a *B. pseudomallei* genotype that was also present in their drinking water (Table 2).

**Table 2:** Genotyping of *Burkholderia pseudomallei* isolated from clinical specimens and water samples for 2 patients, Thailand, 2012*

Patient no./ age, y/sex	Sample type	No. colonies tested	PFGE pattern (no. colonies)	No. bands different from clinical sample	Sequence type determined by MLST	Clinical features and outcome
1/78/F	Blood	1	A (1)	NA	208	No known risk factors for melioidosis; patient had acute onset of severe pneumonia and septic shock and died 48 h after hospital admission. Blood, sputum, and urine specimens were positive for *B. pseudomallei*. Abdominal ultrasound scan was not performed. Patient had a history of consuming untreated well water.
	Well water	10	A (2)	0	208	
			B (2)	8	54	
			C (4)	11	58	
			D (2)	12	309	
2/88/M	Blood	1	E (1)	NA	48	Patient had diabetes, acute onset of pneumonia, and right hemiparesis. Blood and sputum specimens were positive for *B. pseudomallei*. Computer tomography scan of the brain showed a ruptured mycotic aneurysm, leading to acute hematoma in the left frontoparietotemporal lobe and multiple septic emboli. Abdominal ultrasonogram was not performed. Patient had a history of consuming untreated well water and tap water. Septic shock developed on day 19 and the patient died on day 24 after hospital admission.
	Tap water	10	F (7)	3	48	
			G (2)	6	47	
			H (1)	14	696	
					

*Genotyping was performed by using pulsed-field gel electrophoresis (PFGE) and multilocus sequence typing (MLST) as described (*7**,**8*). NA, not applicable.

## Conclusions

Our finding that drinking water, including public tap water, in northeastern Thailand contains viable *B. pseudomallei* is a public health concern. Public tap water in Thailand, to which melioidosis is highly endemic, needs to be safe and free from *B. pseudomallei* contamination. *B. pseudomallei* can survive in water for prolonged periods (*9*). The National Tap Water Quality Assurance Program in Thailand currently does not include *B. pseudomallei* detection (*10*) and this fact warrants review. Unlike observations in Hong Kong (*11*), all collected rainwater specimens were culture negative for *B. pseudomallei*. The reasons for this finding are not known, but it may be that the bacterial count was below the detection limit.

Clusters of melioidosis cases with the same genotype in Australia might be related to a temporary stoppage of the water purification process (*2*,*3*). Clustering has not been reported from Thailand, and might be explained by the high degree of genetic diversity of *B. pseudomallei* in water in this setting (*12*). The validity of a study that reported the detection of *B. pseudomallei* in 6 (7%) of 85 drinking water samples in Italy is questionable (*13*) because the bacterial isolates were not confirmed to be *B. pseudomallei* by using specific identification methods, and there have been no reported cases of indigenous melioidosis in Italy (*14*).

We propose that ingestion was the probable route of melioidosis acquisition in the 2 patients who each had matching *B. pseudomallei* genotypes in their clinical sample and drinking water. Although other routes of infection, such as inoculation injury, are possible for these 2 patients, genetic diversity of *B. pseudomallei* in even a small area in Thailand indicates that the likelihood of observing an identical genotype for an isolate in water and an isolate from a human that was acquired by soil inoculation is probably low (*12*). Infection with >1 strain of *B. pseudomallei* is rare (*15*), but we might not have linked clinical and water strains in some cases because we did not pick appropriate colonies for genotyping from a water sample that contained multiple genotypes. In summary, evidence from a case–control study (*5*) and our molecular study suggests that most *B. pseudomallei* infections are acquired by inoculation, but a proportion of cases are caused by ingestion, and such cases are potentially preventable.
